# Supplementary material for: Clinical Value of Galectin-9, Soluble TREM-1, and Soluble CD25 Among Critically Ill Patients with Organ Failure in the Emergency Department: A Prospective Observational Study
Source: Diagnostics (Basel). 2025 Oct 23;15(21):2677. doi: 10.3390/diagnostics15212677 (PMC12609408; doi:10.3390/diagnostics15212677)
Supplement: Supplementary file 1 [file diagnostics-15-02677-s001.zip › diagnostics-3849656-supplementary.pdf]

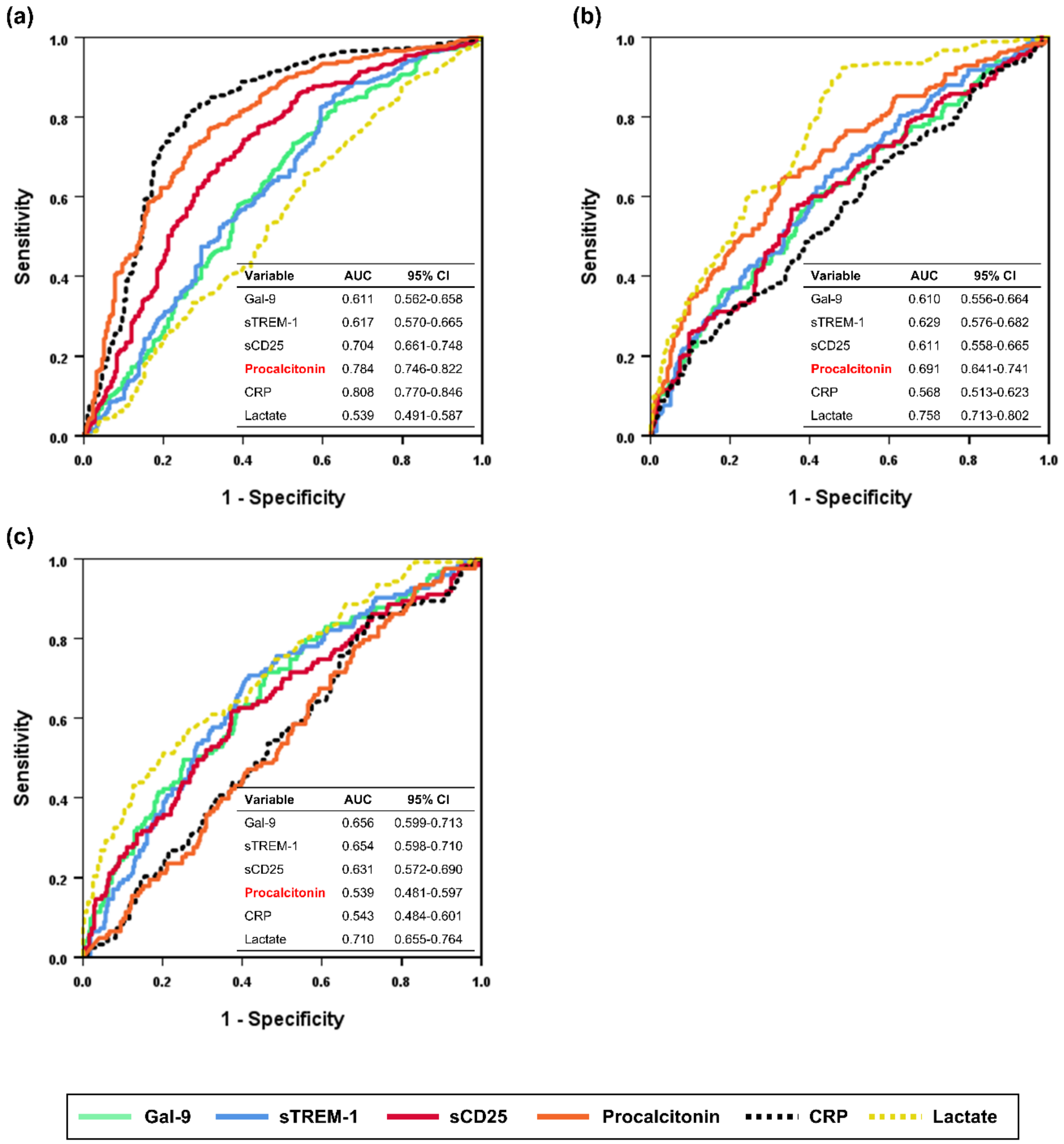

Supplementary Figure S1. ROC curve analyses including procalcitonin **(a)** Discriminating sepsis from NIOF. **(b)** Discriminating septic shock from sepsis. **(c)** Predicting 30-day mortality in sepsis including septic shock. ROC, Receiver operating characteristic; Gal-9, galectin-9; sTREM-1, soluble triggering receptor expressed on myeloid cells-1; sCD25, soluble CD25; CRP, C-reactive protein; NIOF, non-infectious organ failure.

1. Discriminating sepsis from NIOF. (Analysis including Procalcitonin)

Biomarker	AUC	95% CI	p-value
Galectin-9	0.611	0.562–0.658	< 0.001
sTREM-1	0.617	0.570–0.665	< 0.001
sCD25	0.704	0.661–0.748	< 0.001
Procalcitonin	0.784	0.746–0.822	< 0.001
CRP	0.808	0.770–0.846	< 0.001
Lactate	0.539	0.491–0.587	0.101

2. Discriminating septic shock from sepsis. (Analysis including Procalcitonin)

Biomarker	AUC	95% CI	p-value
Galectin-9	0.610	0.556–0.664	< 0.001
sTREM-1	0.629	0.576–0.682	< 0.001
sCD25	0.611	0.558–0.665	< 0.001
Procalcitonin	0.691	0.641–0.741	< 0.001
CRP	0.568	0.513–0.623	0.015
Lactate	0.758	0.713–0.802	< 0.001

3. Predicting 30-day mortality in sepsis. (Analysis including Procalcitonin)

Biomarker	AUC	95% CI	p-value
Galectin-9	0.656	0.599–0.713	< 0.001
sTREM-1	0.654	0.598–0.710	< 0.001
sCD25	0.631	0.572–0.690	< 0.001
Procalcitonin	0.539	0.481–0.597	0.204
CRP	0.543	0.484–0.601	0.163
Lactate	0.710	0.655–0.764	< 0.001
